# Comparison of p53 and the PDZ domain containing protein MAGI-3 regulation by the E6 protein from high-risk human papillomaviruses

**DOI:** 10.1186/1743-422X-5-67

**Published:** 2008-06-02

**Authors:** Julia Ainsworth, Miranda Thomas, Lawrence Banks, Francois Coutlee, Greg Matlashewski

**Affiliations:** 1 Department of Microbiology and Immunology, McGill University, Montreal, QC., Canada; 2 International Centre for Genetic Engineering and Biotechnology, Padriciano 99, Trieste I-34012, Italy; 3 Department de Microbiologie et Immunologie, University de Montreal, QC., Canada; 4 Department of Microbiology and Immunology, McGill University, Montreal, H3A 2B4, 514-398-3914, Canada

## Abstract

Central to cellular transformation caused by human papillomaviruses (HPVs) is the ability of E6 proteins to target cellular p53 and proteins containing PDZ domains, including MAGI-3, for degradation. The aim of this study was to compare E6-mediated degradation of p53 and MAGI-3 under parallel experimental conditions and further with respect to the involvement of proteasomes and ubiquitination. We also compared the degradation of p53 and MAGI-3 by E6 from several HPV types including different variants from HPV-33. All of the E6 genes from different HPV types displayed similar abilities to mediate the degradation of both p53 and MAGI-3 although there may be subtle differences observed with the different 33E6 variants. There were however differences in E6 mediated degradation of p53 and MAGI-3. Proteasome inhibition assays partially protected p53 from E6 mediated degradation, but did not protect MAGI-3. In addition, under conditions where p53 was ubiquitinated by E6 and MDM2 *in vivo*, ubiquitination of MAGI-3 was not detected. These results imply that although both p53 and MAGI-3 represent effective targets for oncogenic E6, the mechanisms by which E6 mediates p53 and MAGI-3 degradation are distinct with respect to the involvement of ubiquitination prior to proteasomal degradation.

## Background

Over 100 types of human papillomaviruses (HPVs) have been identified and they represent etiological agents for conditions ranging from benign warts to cervical cancer. Approximately 18 of the known HPV types are classified as high-risk due to their association with anogenital cancers and low-grade to high-grade dysplasias [1]. High-risk HPV types 16 and 18 represent the most extensively studied high-risk HPV types and together account for approximately 70% of cervical cancers worldwide, while the other high-risk HPV types are responsible for the remainder [2].

High-risk HPV types derive their oncogenicity primarily from the E6 and E7 transforming proteins (reviewed in [3]). E7 leads to the constitutive activation of cellular proliferation genes principally via release of the E2F transcription factor from the retinoblastoma tumor suppressor protein, pRb. The E6 protein inhibits cellular apoptosis by inactivating p53 predominantly via proteasome-mediated degradation. In uninfected cells, p53 is principally regulated by the cellular E3 ubiquitin ligase MDM2 which targets p53 for ubiquitin-mediated proteasomal degradation (reviewed in [4]). Contrarily, in HPV-positive cancer cells, the MDM2 degradation pathway is non-functional. HPV E6 proteins, however, associate with the cellular E6 associated protein (E6AP) which ubiquitinates p53 primarily in the nucleus, thus targeting E6 for proteasomal degradation in both the nucleus and cytoplasm [5,6]. More recent studies have shown that E6 can also mediate loss of p53 activity through mechanisms independent of E6AP and ubiquitination [7-9].

Another less-understood target of HPV is the family of PDZ domain-containing cellular proteins. PDZ domains consist of 80–90 amino acids and are amongst the most common protein-protein interaction domains found in human cells (reviewed in [10]). PDZ domains are often present in transmembrane receptors, channel proteins, and/or other PDZ domains and appear to function as scaffolds for the assembly of supra-molecular complexes important in signaling, cell-cell adhesion, ion transport, and formation of tight junctions [11]. PDZ proteins are grouped based on structure, with the largest group being the MAGUK family, which generally contains 1–6 PDZ domains and a characteristic inactive guanylate kinase-like domain at the C-terminus [12]. MAGUK members may be important in tumor suppression, organization of signaling complexes, and membrane protein trafficking [13]. MAGUK is further divided into subfamilies, one of which is distinguished by an N-terminal GUK domain and, as such, is known as MAGUK inverted (MAGI). There are three MAGI proteins, specifically MAGIs 1–3. MAGIs 1 and 3 exhibit widespread tissue expression, but tend to localize to tight junctions between epithelial cells [14]. MAGI-2, on the other hand, appears to be explicitly neuronal and required during development [15]. The precise functions of MAGI proteins are unknown; however, all MAGI proteins have been shown to bind the PTEN tumor suppressor, whose PDZ-binding domain is important for its tumor suppressor function [16-18].

The HPV E6 protein is able to target various PDZ domain-containing proteins for degradation including, hDlg, hScrib, MUPP-1, and MAGIs 1–3 [19-22]. Only high-risk HPV E6 proteins containing the C-terminal sequence X-T/S-X-V/L can interact with PDZ domain-containing proteins, and mediate their degradation [23] and this process appears to be necessary for cell transformation [24]. Recent studies have demonstrated that E6 uses both E6AP-dependent and E6AP-independent mechanisms, to mediate the degradation of different PDZ domain-containing proteins [7,25]. Regardless of whether E6AP is involved, the role of MAGI ubiquitination *in vivo *during E6-mediated proteasome degradation has not been resolved.

Since p53 and MAGI-3 represent distinct targets for high risk HPV E6, our approach was to directly compare p53 and MAGI-3 degradation by E6 from several high risk HPV types and further, to compare E6-mediated ubiquitination of p53 and MAGI-3. The results of this study provide a better understanding about the interactions of viral E6 with key cellular regulatory proteins.

## Results

### Comparison of p53 and MAGI-3 degradation by high risk HPV-E6

**Figure 1:**
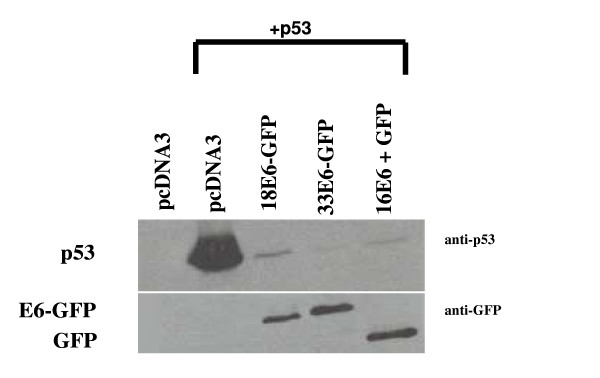
Western blot analysis of p53 degradation in the presence of HPV-18 E6-GFP, HPV-33 E6-GFP and HPV-16 E6. Cells were transfected with plasmids expressing pcDNA3 (control), p53, and p53 co-transfected with plasmids expressing HPV-18 E6-GFP, HPV-33 E6-GFP, or HPV-16 E6 + GFP as indicated. Upper panel: Western blot analysis of p53. Lower panel: Western blot analysis of E6-GFP and non-fused GFP.

We have previously demonstrated that HPV-18 E6 and E6-GFP fusion proteins were equally active at mediating p53 degradation in transfected cell lines [26]. The fusion of GFP to the N-terminal of E6 therefore enabled the detection of E6-GFP by immunofluoresence and Western blot analysis using anti-GFP antibodies since antibodies to E6 are not available. In the first experiment, we compared p53 degradation in the presence of 18E6-GFP and 33E6-GFP *in vivo *in p53-null H1299 cells. For comparison, we also included a co-transfection with a plasmid encoding the wildtype HPV-16 E6 protein plus a plasmid expressing free GFP. As shown in Figure 1 (upper panel), HPV-18 E6-GFP and HPV-33 E6-GFP fusion proteins mediated similar levels of p53 degradation. Likewise, co-transfection of two plasmids expressing HPV-16 E6 and GFP separately also mediated p53 degradation to similar levels as the HPV type 18 and 33 E6-GFP fusion proteins. In this manner, it was possible to show similar levels of E6-GFP fusion proteins and free GFP in these transfected cells (Fig. 1, lower panel). Since no lower molecular weight degradation products were detected on this Western blot with the anti-GFP antibodies, this suggests that the E6-GFP fusion proteins remained intact in the transfected cells. In the presence of similar levels of E6-GFP (lower panel), there were also similar levels of p53 remaining following E6-mediated p53 degradation (Upper panel). This experiment therefore revealed comparable effectiveness between HPV types 16, 18, and 33 E6 at mediating the degradation of p53 *in vivo *and showed that GFP can be used as an effective epitope tag for comparing E6 levels in transfected cells.

**Figure 2:**
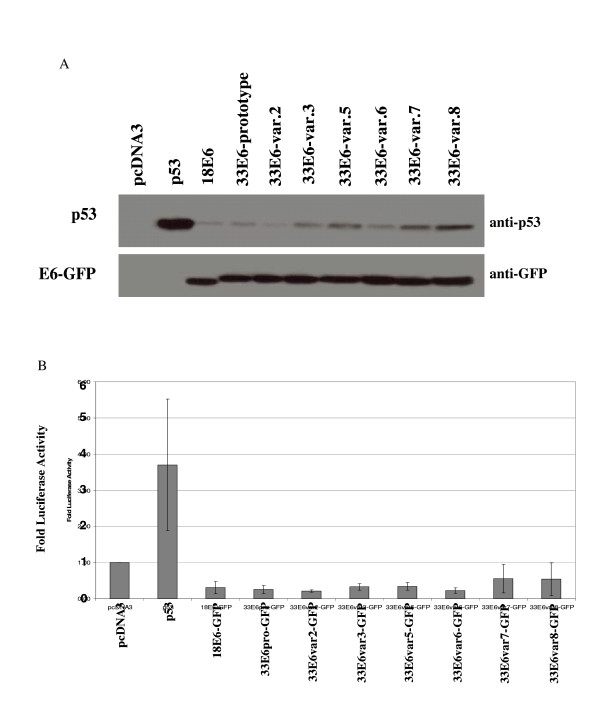
Comparing p53 protein levels and p53 transcriptional activity in cells expressing E6-GFP from HPV types 18, 33, and 33 variants. Panel A: Western blot analysis of p53 and E6-GFP. Panel B: p53 transcriptional activity as determined by measuring luciferase activity in cells co-transfected with the p53 responsive p21-luciferase reporter plasmid.

Impairment of p53 activity may not be directly proportional to its degradation because E6 impairs p53 initially by directing its nuclear export and subsequently mediating the majority of p53 degradation in the cytoplasm [5]. We thereby assayed for p53-mediated transcriptional activity in the presence of E6 from HPV-18 and HPV-33. In addition, a number of HPV-33 variant viruses have been identified from infected individuals where the E6 proteins differ by one or several amino acids, as shown in Table 1[27]. It was therefore interesting to determine whether polymorphisms in these HPV type 33 E6 genes affect their ability to mediate loss of p53 activity. For this analysis, E6-GFP fusion proteins from HPV-18 E6, prototype HPV-33 E6, and several variants of HPV-33 E6 were co-transfected with plasmids expressing p53 and a p53-responsive p21 luciferase reporter plasmid. Cell lysates were then prepared for the measurement of luciferase activity, and later assessed by Western blot analysis to determine p53 and E6-GFP levels. The result of the Western blot can be seen in Figure 2A, which shows that all of the constructs expressing the various E6-GFP fusion proteins mediated p53 degradation to various degrees relative to the p53 control (no E6). The expression levels of the different E6-GFP gene products were virtually the same for each transfection making it possible to accurately compare their ability to mediate p53 degradation under the same conditions in the presence of the same amount of substrate p53. It is noteworthy that it was possible to detect different p53 levels in the presence and absence of the various E6s and therefore comparisons between the various E6s could be made. This Western blot suggested that some variants may be more effective than others at mediating p53 degradation. For example, HPV-33 E6 variant 2 appeared to be more effective at mediating p53 degradation than HPV-33 E6 variants 7 and 8. The E6-mediated impairment of p53 transcriptional activity in these transfected cells can be seen in Figure 2B. It is also noteworthy that HPV-33 E6 variants 2 and 6 reduced p53 activity to a greater extent than did HPV-33 E6 variants 7 and 8, consistent with the corresponding Western blot. Taken together, these results show that HPV-33 E6 and the different HPV-33 E6 variants all mediated the impairment of p53 transcriptional activity to a similar extent as HPV-18 E6 but that some HPV-33 variants may be more efficient than others at degrading p53.

**Table 1:** Sequence polymorphisms in the HPV-33 E6 variants

**Codon Position**	**HPV-33 E6 Variants**	**Nucleotide Change**	**Amino Acid Change**
18	5	C to T	A to V
28	8	T to G	L to R
36	6, 7, 8	A to C	K to N
36	3, 5	C to A	P to T
69	2, 3, 5	C to T	F to F (none)
73	2, 3, 5	A to C	I to L
83	2, 3, 5	G to T	V to L
86	7	A to C	N to H
93	2, 3, 5	A to C	K to N
125	7	A to T	R to R (none)
138	2, 3, 5	C to T	A to V
142	2, 3, 5	C to T	S to S (none)

Codon positions of polymorphisms pertaining to the HPV-33 E6 variants. Also indicated are the specific changes in nucleotide and amino acid sequence from that of the HPV-33 E6 prototype (i.e. prototype nucleotide/amino acid to polymorphic nucleotide/amino acid).

**Figure 3:**
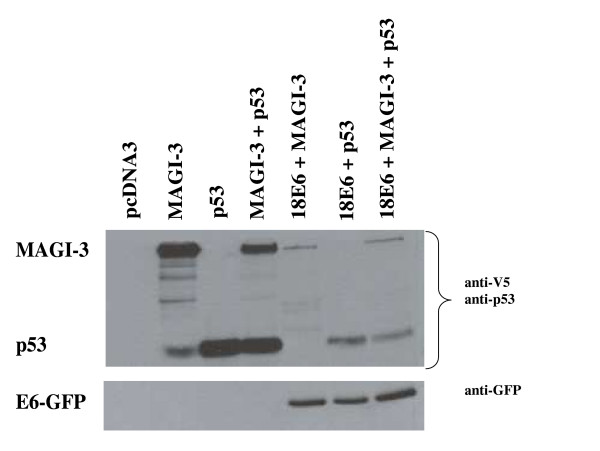
Western blot analysis of p53 and MAGI-3 levels expressed separately or together in the presence of HPV-18 E6-GFP as indicated. Upper panel: Western blot analysis with antibodies against p53 or MAGI-3 (anti-V5 tag). Lower panel: Western blot analysis of E6-GFP. Note that the level of E6 mediated p53 and MAGI-3 degradation is very similar and do not compete in the presence of E6.

It is clear that E6 proteins from high-risk HPV types mediate the degradation of both p53 and several PDZ domain-containing proteins, including MAGI-3. However, since these cellular proteins perform different functions, it is interesting to know whether E6-mediated loss of p53 and MAGI-3 with equal efficiency and whether this is carried out in a similar manner in the cell. We therefore compared the degradation of p53 and MAGI-3 separately and simultaneously (Fig. 3, upper panel) under assay conditions, where there were equal levels of transfected p53 and E6 protein in the p53 null H1299 cells (Figure 3, lower panel). It has been reported that endogenous MAGI-3 is undetectable in cell lines suggesting that it is in low or undetectable levels in these cells [28]. Under these experimental conditions it was therefore possible to compare the levels of transfected p53 and MAGI-3 in the presence and absence equal amounts of transfected E6. The results from this experiment suggested that, when assayed under the same conditions, 18E6 mediated degradation of p53 and MAGI-3 to similar extents (Figure 3).

**Figure 4:**
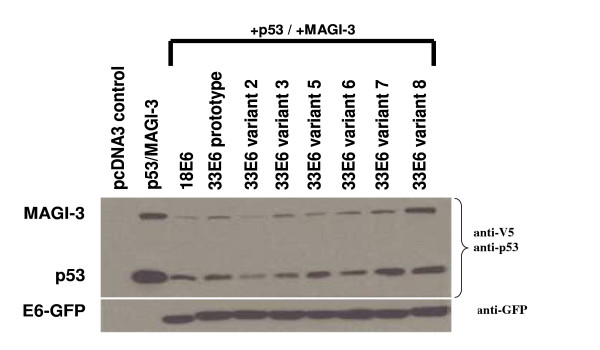
Western blot analysis of p53 and MAGI-3 levels in the presence of HPV type 18 E6-GFP, type 33 E6-GFP and type 33 variants E6-GFP as indicated. Upper panel: Western blot analysis of p53 and MAGI-3 (anti-V5 antibodies). Lower panel: Western blot analysis of E6-GFP. Note that HPV-33 E6 variant 2 was the most effective at mediating the degradation of both p53 and MAGI-3 and HPV-33 E6 variants 7 and 8 were the least effective.

Since some of the HPV-33 E6 variants appeared to be more efficient than others at mediating p53 degradation, as shown in Figure 2, it was of interest to compare their ability to mediate the degradation of MAGI-3. Moreover, as shown in Table 2, the C-terminal PDZ binding domain for HPV-33 E6 is not well conserved compared to HPV type 18 E6. This may therefore suggest that HPV type 33 E6 may not be as effective as HPV type 18 E6 at mediating MAGI-3 degradation. We therefore compared the degradation p53 and MAGI-3 in the presence of the HPV-33 E6 prototype and several of its variants. H1299 cells were co-transfected with p53 and MAGI-3 expression plasmids along with constructs encoding pcDNA3 (control), HPV-18 E6-GFP, the HPV-33 E6-GFP prototype, and the different HPV-33 E6-GFP variants. As shown in Figure 4 (upper panel), HPV-33 E6 and its variants were effective at mediating MAGI-3 degradation. Variant 2 was the most active while variants 7 and 8 appeared to be the least active at mediating both MAGI-3 and p53 degradation. Notably, this is consistent with the results shown in Figure 2 (with respect to p53) showing reproducibility in these transfection assays. Western blot analysis of the different transfection-derived E6-GFPs confirmed that they were present in equal amounts (lower panel). These results suggest that E6 proteins that were more efficient at mediating p53 degradation are also more efficient at mediating MAGI-3 degradation.

**Table 2:** C-terminal PDZ sequences for different HPV types

**HPV Type (E6)**	**PDZ domain**								
18	**L**	**Q**	**R**	**R**	**R**	**E**	**T**	**Q**	**V**
45	**L**	R	**R**	**R**	**R**	**E**	**T**	**Q**	**V**
70	R	R	I	**R**	**R**	**E**	**T**	**Q**	**V**
58	R	P	**R**	**R**	**R**	Q	**T**	**Q**	**V**
16	S	R	T	**R**	**R**	**E**	**T**	**Q**	L
35	K	P	T	**R**	**R**	**E**	**T**	E	**V**
51	T	R	Q	**R**	N	**E**	**T**	**Q**	**V**
82	A	R	Q	**R**	S	**E**	**T**	**Q**	**V**
33	R	S	**R**	**R**	**R**	**E**	**T**	A	L
56	S	R	E	P	**R**	**E**	S	T	**V**
66	S	R	Q	A	T	**E**	S	T	**V**
53	H	T	T	A	T	**E**	S	A	**V**

Comparison of the C-terminal PDZ-binding domains for E6 from high-risk HPV types relative to HPV-18 E6. Amino acid sequences homologous to HPV-18 are highlighted in boldy and amino acids matching the consensus PDZ-binding sequence X-T/S-X-V/L are underlined.

### Comparison of E6-mediated ubiquitination and proteasome degradation of p53 and MAGI-3

**Figure 5:**
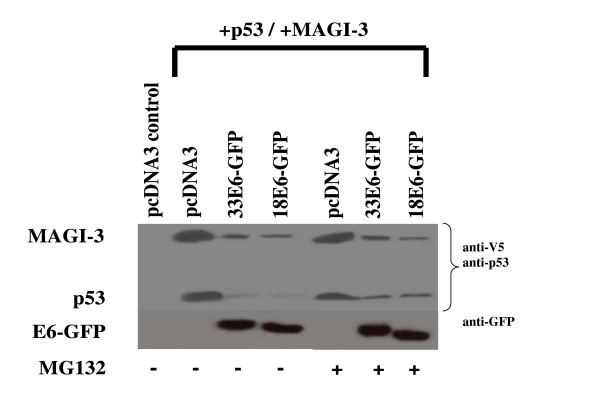
p53 and MAGI-3 protein levels in cells expressing HPV-18 E6 and HPV-33 E6 following proteasome inhibition. Cells were transfected with plasmids expressing p53, MAGI-3, and E6-GFP fusion proteins in the presence and absence of proteasome inhibition with MG132 as indicated. Upper panel: Western blot analysis of p53 and MAGI-3 (anti-V5 tag). Lower panel: Western blot analysis of E6-GFP. Note that addition of MG132 partially restored p53 levels but not MAGI-3 levels.

It has recently been established that E6 directs the degradation of p53 by both ubiquitin-mediated proteasome-dependant and -independent pathways *in vivo *[5,8]. Ubiquitin-mediated proteasome degradation has not been established for E6-mediated degradation of MAGI-3 *in vivo*. The preceding experiments showed a close correlation between p53 and MAGI-3 degradation by E6. It was therefore of interest to compare E6-mediated proteasomal degradation and ubiquitination of p53 and MAGI-3. p53 and MAGI-3 expression plasmids were initially transfected in H1299 cells along with pcDNA3 (control), HPV-18 E6-GFP, or the HPV-33 E6-GFP prototype both in the presence and absence of the proteasome inhibitor MG132. Although the half life of the ectopically expressed p53 and MAGI-3 in the transfected cells is not known, the amount of plasmid derived p53 and MAGI-3 was approximately the same 24 hrs following transfection in the absence of E6 (Fig. 5, upper panel, lanes 2 and 5). In the presence of E6, the level remaining p53 and MAGI-3 was similar (Fig. 5, upper panel, lanes 3 and 4) suggesting that, under these conditions, there was a similar rate of E6 mediate degradation of the transfected p53 and MAGI-3. There was about a 2 fold increase in the amount of p53 after 4 hrs treatment with MG132 (Fig. 5 upper panel, Lanes 5 and 6) relative to cells not treated with MG132 (Fig. 5 upper panel, Lanes 3 and 4) in the cells co-transfected with E6. In contrast, under the same conditions, there was no similar increase in the stability of MAGI-3 in the presence of MG132 relative to the non-treated cells. This demonstrated that E6 mediated degradation of p53 was more sensitive to proteasome inhibition than E6 mediated degradation of MAGI-3 and this was observed for both types 18 and 33 E6. Western blot analysis confirmed equal levels of HPV-18 E6-GFP and HPV-33 E6-GFP levels in these assays (lower panel).

**Figure 6:**
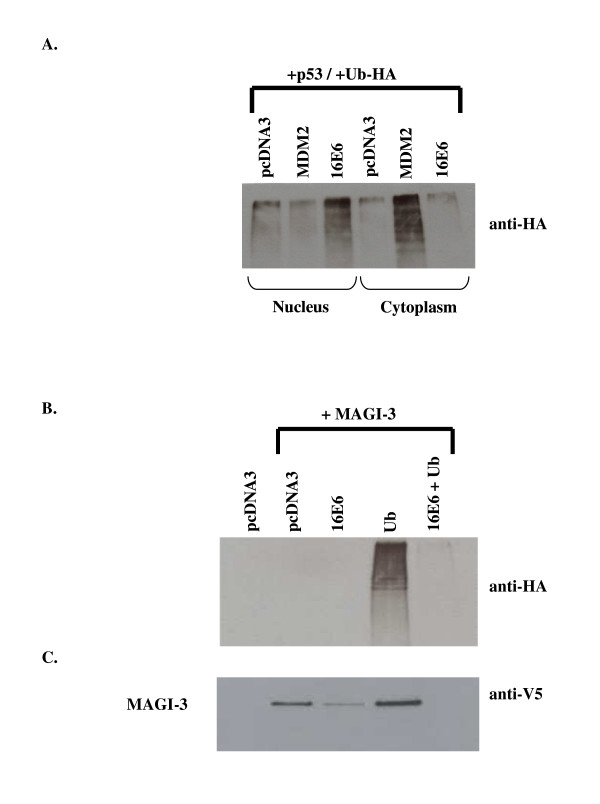
Comparison of E6-mediated ubiquitination of p53 and MAGI-3. Panel A. p53 ubiquitination in nuclear and cytoplasmic extracts from cells transfected with plasmids expressing pcDNA3 (control), MDM2 and HPV-16 E6 as indicated. Panel B. MAGI-3 ubiquitination from cells transfected with plasmids expressing pcDNA3 (control), HPV-16 E6, HA-tagged ubiquitin (Ub) alone, or HPV-16 E6 + HA-tagged ubiquitin (Ub). Panel C. MAGI-3 protein levels in cells transfected with plasmids expressing pcDNA3 (control), HPV-16 E6, HA-tagged ubiquitin (Ub) alone, or HPV-16 E6 + HA-tagged ubiquitin (Ub).

Based on these observations, we next compared the ability of E6 to mediate the ubiquitination of p53 and MAGI-3, which is often a precursor to proteasome-mediated degradation. Initially we established the experimental conditions for p53 ubiquitination in the presence of E6 and MDM2. H1299 cells were transfected with plasmids expressing p53 and HA epitope-tagged ubiquitin in the presence of either pcDNA3 (control), HPV-16 E6, or MDM2. The proteasome inhibitor MG132 was added 4 hours prior to preparing the cell extracts to stabilize ubiquitinated p53. Preparation of both nuclear and cytoplasmic extracts was followed by immunoprecipitation of p53, and ultimately, Western blotting with anti-HA antibodies to detect ubiquitinated p53. As shown in Figure 6A, E6 mediated p53 ubiquitination predominately in the nucleus while MDM2 mediated p53 ubiquitination predominately in the cytoplasm, consistent with our previous observations [5].

Using the same experimental conditions, we examined the ubiquitination of MAGI-3 in the presence and absence of HPV-16 E6. H1299 cells were transfected with plasmids expressing MAGI-3 in the presence of either pcDNA3 (control), HPV-16 E6, HA epitope-tagged ubiquitin alone, or HPV-16 E6 plus HA epitope-tagged ubiquitin together. Total cell lysates were prepared following a 4 hour treatment with the proteasome inhibitor MG132 to stabilize ubiquitinated intermediates. Only cytoplasmic extracts were prepared since MAGI-3 is predominantly a cytoplasmic protein. Immunoprecipitation of MAGI-3 with anti-V5 antibody was followed by Western blot analysis with anti-HA antibody to detect ubiquitinated MAGI-3. As shown in Figure 6B, MAGI-3 ubiquitination was clearly detectable in the absence of E6. In the presence of E6, there was a sharp reduction in detectable ubiquitinated MAGI-3. Figure 6C reveals that E6 mediated MAGI-3 degradation despite the reduction in detectable MAGI-3 ubiquitination in the presence of E6 observed in Figure 6B. Therefore, under conditions where E6 mediated the degradation of both p53 and MAGI-3, there is in increase in detectable p53 ubiquitination and a decrease in detectable MAGI-3 ubiquitination.

## Discussion

Previous studies have demonstrated the ability of E6 from high-risk HPV types to target p53 and PDZ domain-containing proteins, including MAGI-3, for cell-mediated degradation (reviewed in [3]). However, it is not known whether E6 targets p53 and MAGI-3 with equally efficiency under identical conditions and whether E6 mediates the ubiquitination of MAGI-3 *in vivo *similar to p53. We have begun to address these questions in the present study toward developing a better understanding of HPV-host cell interactions. We first examined whether E6 preferentially mediated the degradation of p53 or MAGI-3 when both were co-expressed in the presence of E6. There were two notable outcomes to this analysis. First, the levels of p53 and MAGI-3 degradation were similar when both proteins were co-expressed in the presence of HPV-18 E6. Second, comparison of a panel of HPV-33 E6 variants suggested that the E6 variants, which were more effective at mediating p53 degradation, were also more effective at mediating MAGI-3 degradation.

Although, the prototype HPV-33 E6 is as effective as HPV-18 E6 at targeting p53 and MAGI-3 *in vivo*, it was interesting to note that HPV-33 E6 variant 2 was consistently more active than the prototype and additionally, that HPV-33 E6 variants 7 and 8 appeared to be the least active. This observation was further supported by measuring the inhibition of p53-mediated transcription. Interestingly, HPV-33 E6 variant 2 has four polymorphic changes in amino acid sequence which are also present in variants 3 and 5; however, variants 3 and 5 contain the amino acid change P36T while variant 2 does not. This implies that P36T may actually decrease the p53 and MAGI-3 degradative abilities of variants 3 and 5 relative to variant 2. Further, the variant 2 polymorphisms are located outside of the C-terminal consensus-binding site for PDZ domain-containing proteins suggesting that sequences outside of the C-terminal can have direct or indirect influence on the ability to mediate MAGI-3 degradation. Although HPV-33 E6 variant 2 was the most effective at mediating degradation of p53 and MAGI-3, it does not appear to be associated with an increased risk of high-grade disease although studies involving larger populations of HPV-33 carriers are needed to confirm this [27].

We also compared E6-mediated degradation of p53 and MAGI-3 in the presence of the proteasome inhibitor MG132. Under conditions where p53 levels were partially restored by proteasome inhibition, MAGI-3 levels were the same in both the absence and presence of MG132. To further examine this difference, we compared E6-mediated ubiquitination of p53 and MAGI-3 since ubiquitination is often a precursor to proteasome-mediated degradation. We observed that MAGI-3 ubiquitination was detectable in the absence of E6 and that the level of detectable MAGI-3 ubiquitination was dramatically reduced in the presence of E6. This suggest that, if E6 did mediate ubiquitination of MAGI-3 prior to proteasome degradation, it did so more rapidly than for p53 since E6 and MDM ubiquitinated p53 intermediates were detectable under these conditions. Alternatively, E6 was able to mediate MAGI-3 degradation in an ubiquitin independent manner as recently described for p53 which is degraded by both ubiquitin dependent and independent mechanisms [8]. This explanation would be consistent with the observation shown in Figure 5 that impairment of ubiquitin mediated proteasome degradation with MG132 partially protected p53 but not MAGI-3.

## Conclusion

One of the major advantages of this study has been the ability to compare the degradation of p53 and MAGI-3 under conditions where E6 levels can be directly compared using antibodies to the GFP tag. In this manner, it was possible to rule out the possibility that differences in target protein degradation levels were due to differences in transfected E6 levels. It was interesting to note that those HPV-33 E6 variants, which appeared to be more efficient at mediating p53 degradation, also appeared to be more efficient at mediating MAGI-3 degradation. Consequently, polymorphisms in HPV-33 E6 may have evolved to maintain a balance between the ability to degrade p53 and MAGI-3, suggesting that as the level of p53 is reduced in the infected cell, it is also necessary to reduce MAGI-3 levels. Future studies are now needed to determine the involvement of E6-mediated ubiquitination of MAGI-3 *in vivo *since it is likely through a different mechanism than E6-mediated p53 ubiquitination.

## Methods

### Cell lines and Transfections

Human p53-null H1299 epithelial cells, kindly provided by Dr. P. Branton (McGill University), were used in this study. Cells were cultured in Dulbecco's modified Eagle's medium (DMEM) (GIBCO) with 10% fetal bovine serum (FBS) (GIBCO) and 100 units penicillin-streptomycin ml^-1 ^(GIBCO). Cells were transfected with Lipofectamine (GIBCO) according to the manufacturer's protocol and cell lysates were harvested 24 h post-transfection.

### Construction of E6-GFP Fusion Proteins

The HPV-33 E6-GFP fusion proteins, containing GFP at the N-terminus, were generated by amplifying their respective E6 sequences out of previous E6 gene containing vectors [27] using PCR primers (Alpha DNA) including Bgl II (5') and EcoRI (3') restriction sites. The upstream primer sequence was 5'CAGATCTCATGTTTCAAGACACTGAGGAAAAACCAC while the downstream primer sequence was 5'CAGAATTCGTCACAGTGCAGTTTCT-CTACGTCGG. The amplified E6 sequences were ligated between the Bgl II and EcoRI restriction sites in the multiple cloning site of the pEGFP-C3 vector (Clontech). The HPV-18 E6-GFP fusion protein construct was engineered as previously described [26].

### Detection of p53, MAGI-3, E6, and E6-GFP Fusion Proteins by Western Blot Analysis

H1299 cells were transfected with plasmids expressing p53, MAGI-3 containing a C-terminal V5 epitope tag, and control pcDNA3.1, HPV-E6, or HPV E6-GFP expression vectors essentially as previously described [5,26]. 24 h post-transfection, cells were washed with cold phosphate-buffered saline (PBS) and harvested on ice in cold lysis buffer (50 mM Tris-HCl, pH 8.0; 150 mM NaCl, 1% NP40, protease inhibitor cocktail (Roche)). Cell debris was eliminated via centrifugation at 14 000 rpm for 10 min at 4°C. Lysates were boiled in 1.5× SDS-PAGE sample buffer (45 mM Tris-HCl, pH6.8; 10% glycerol, 2% SDS, 5% β-mercaptoethanol, 0.005% bromphenol blue). Lysates were resolved on a 10% SDS-PAGE gel. Following transfer of the separated proteins to a nitrocellulose membrane (Bio-Rad Laboratories), a Western blot analysis was performed. The membrane was probed with primary monoclonal antibodies DO-1, (1:5000) (Calbiochem) for detection of p53 levels, and V5, (1:2500) (Invitrogen) for detection of exogenous MAGI-3 levels. The membrane was subsequently incubated with anti-mouse IgG HRP (horseradish peroxidase)-linked antibody (1:7000) (Amersham Pharmacia). The proteins were visualized using the enhanced chemiluminescence (ECL) detection system (Amersham) according to the manufacturer's instructions. The membrane was then stripped and re-probed for GFP (to detect E6-GFP) using mAb JL-8 antibody (1:5000) (Clontech).

### Proteasome Inhibition Assay

The protocol was performed exactly as described above, except the addition of 10 uM MG132 proteasome inhibitor (Calbiochem) was added for 4 hours at 20 h post-transfection.

### MAGI-3 and p53 Ubiquitination Assay

As previously detailed [5], H1299 cells were transfected with a plasmid expressing MAGI-3 in the presence of control pcDNA3.1, HPV-16 E6, or a hemaglutinin (HA)-tagged ubiquitin expression plasmid. At 20 h post-transfection, 20 uM MG132 proteasome inhibitor (Calbiochem) was added. At 24 h, cells were harvested and total cell lysates were collected in cold lysis buffer (50 mM Tris-HCl, pH 8.0; 150 mM NaCl, 1% NP40, 5 mM NEM) protease inhibitor cocktail (Roche)). Cell debris was eliminated via centrifugation at 14 000 rpm for 10 min at 4°C. Lysates were subjected to overnight immunoprecipitation with αV5 mAb against exogenous MAGI-3 (1:1000) (Invitrogen) at 4°C, followed by the addition of a 1/10^th ^volume of protein A-sepharose beads (Sigma) for 30 min at 4°C. Immunoprecipitates were washed four times with cold HB buffer (10 mM Tris-HCl, pH 1.9; 1.5 mM MgCl_2_, 1 M KCl, protease inhibitor cocktail), resolved via SDS-PAGE (8%), and ultimately analyzed by Western blot using a mouse monoclonal anti-HA HRP-conjugated antibody (1:5000) (Roche) to detect ubiquitinated MAGI-3.

## Authors' contributions

JA carried out the experiments shown in figures 1 through 6 under the technical direction of MT, LB, and GM who also participated in the design of the study, data analysis and writing the manuscript, FC provided the information contained in Table 1 and direction on the analysis of the type 33 E6 variants. All authors read and approved of the final manuscript
